# Reverse Mode Polymer Stabilized Cholesteric Liquid Crystal Flexible Films with Excellent Bending Resistance

**DOI:** 10.3390/molecules29174276

**Published:** 2024-09-09

**Authors:** Ping Yu, Zemin He, Yuzhen Zhao, Wenqi Song, Zongcheng Miao

**Affiliations:** 1 Technological Institute of Materials & Energy Science (TIMES), Xi’an Key Laboratory of Advanced Photo-Electronics Materials and Energy Conversion Device, School of Electronic Information, Xijing University, Xi’an 710123, China; yupingtina1128@126.com (P.Y.); zyz19870226@163.com (Y.Z.); songwenqi0123@163.com (W.S.); 2 School of Artificial Intelligence, Optics and Electronics (IOPEN), Northwestern Polytechnical University, Xi’an 710072, China

**Keywords:** cholesteric liquid crystal, reverse-mode, polymer film, spacer columns

## Abstract

The reverse-mode smart windows, which usually fabricated by polymer stabilized liquid crystal (PSLC), are more practical for scenarios where high transparency is a priority for most of the time. However, the polymer stabilized cholesteric liquid crystal (PSCLC) film exhibits poor spacing stability due to the mobility of CLC molecules during the bending deformation. In this work, a reverse-mode PSCLC flexible film with excellent bending resistance was fabricated by the construction of polymer spacer columns. The effect of the concentration of the polymerizable monomer C6M and chiral dopant R811 on the electro-optical properties and polymer microstructure of the film were studied. The sample B2 containing 3 wt% of C6M and 3 wt% R811 presented the best electro-optical performance. The electrical switch between transparent and opaque state of the flexible PSCLC film after bending not only indicated the excellent electro-optical switching performance, but also demonstrated the outstanding bending resistance of the sample with polymer spacer columns, which makes the PSCLC film containing polymer spacer columns have a great potential to be applied in the field of flexible devices.

## 1. Introduction

Smart windows with switchable light transmission capabilities are gaining increasing attention [1,2,3]. Generally, functional materials, including hydrogel [4,5], phase change materials [6,7], electrochromic materials [8,9,10], and liquid crystals [11,12,13,14,15,16], have been widely used to develop smart windows. Among them, liquid crystals (LCs) with electric field responsiveness are attracting researchers to study [15,16,17,18,19]. Cholesteric liquid crystals (CLCs) are one of the most promising LCs materials due to their special molecule rearrangement and adaptive properties [16,17,18]. In CLCs, the directors of LC molecules are tuned by a fix angle to form a helical structure driven by the intermolecular forces of chiral molecules [16]. The pitch of the CLCs is the distance between the quasi-nematic layers with the same directors, and the LC molecules experience a complete twist of 2π angle along the helical axis in that condition [17]. Generally, there are two states of the CLCs, i.e., the planar state and the focal cone state, depending on the orientation of their helix axes [19]. In the planar state, the helix axes are oriented perpendicular to the substrate, and the CLCs can selectively reflect circularly polarized light whose wavelength is equal to the pitch length [18]. In the focal cone state, the helix axes are arranged haphazardly, resulting in the scattering of the incident light. Therefore, the optical state of CLCs can be reversibly switched between reflection and scattering by adjusting the ordering of the helix axes, which allows the CLCs to be used in the field of light shutter, OLEDs, smart windows, and anticounterfeiting labels [19,20,21].

Polymer–liquid crystal composites are phase-separated films in which the responsive characteristics of LCs can be well preserved by the polymer phase [22,23,24,25,26,27,28,29,30,31]. The polymer provides the mechanical or structural stabilization to the LCs when the two are homogeneously mixed [23]. Consequently, the polymer–liquid crystal composites are the best candidates for the fabrication of smart widows due to their unique dynamic light modulation capabilities [24,25,26]. Based on the operating modes, polymer–liquid crystal composites can be categorized into two kinds, i.e., normal-mode and reverse-mode [27,28,29]. The composite films with reverse-mode are transparent in the normal and opaque at power-up, which is more practical for the scenarios where transparency is required most of the time [28]. The reverse-mode polymer–liquid crystal composite film usually fabricated by polymer-stabilized liquid crystals (PSLCs), where the monomers amount is less than 10% of total weight [30]. When CLCs are used as the LC phase, the PSLC film also can be called polymer-stabilized cholesteric liquid crystals (PSCLCs) [31,32,33,34,35]. In PSCLCs, the CLCs is a continuous medium, and the optical state of CLCs is stabilized by the small amount of polymer. However, the PSCLC film exhibits poor spacing stability due to the mobility of CLC molecules during the bending deformation [36]. Therefore, the design and preparation of PSCLC with high stability is a critical technical problem to be solved. Kikuch et al. fabricated flexible and bendable LCDs by forming polymer walls in liquid crystal cells [37]. Kawamorita et al. patterned polymer walls in the LC/monomer mixture via utilization of different wettability [38]. By the aggregation control, the flowing of LCs is prevented by the polymer walls. Li et al. prepared a PSLC film with excellent stability of electro-optical properties by constructing polymer walls [39]. Chen et al. prepared a smart window with high stability by introducing epoxy polymer to liquid crystalline composite and contrasting boscage-like morphology [40]. Yoon et al. proposed a single-step dual stabilization method to fabricate a smart window with high stability by constructing polymer partition walls [41].

Although using the polymer walls could fabricate the flexible LC device, the polymer walls are continuous. In this work, a reverse-mode PSCLC flexible film with excellent bending resistance was fabricated by the construction of polymer spacer columns. The interface between polymer columns and LC is less than that of polymer walls. The influence of the polymerizable monomers and chiral dopant concentration on the electro-optical properties and polymer microstructure of the film were studied. A large-scale flexible film with the optimized sample was fabricated by constructing polymer spacer columns between ITO-coated PET film, and the stability of the film in bending state was also examined. The excellent electro-optical switching performance and outstanding bending resistance of the flexible PSCLC film showed broad application prospects in smart windows for light management. 

## 2. Results and Discussion

### 2.1. Effect of the Concentration of C6M

**Figure 1:**
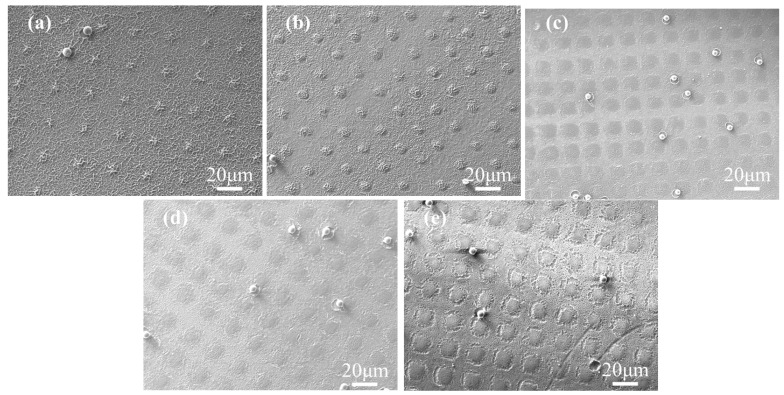
The SEM images of samples A1–A5 with different contents of C6M: (**a**) 1 wt%; (**b**) 2 wt%; (**c**) 3 wt%; (**d**) 4 wt%; (**e**) 5 wt%.

To study the effect of polymerizable monomers, the non-liquid crystal monomers (NLCM) and liquid crystal monomer (LCM) were employed to form the polymer columns. In this system, the monomers with small molecular mass, including PEGDA, HPMA, IBMA and Bis-EMA 15 were used as NLCM, while the C6M with a conjugate structure was used as LCM. The samples A1–A5 with 5 wt% R811 and different C6M contents ranging from 1 wt% to 5 wt% were prepared with UV curing under a photomask. Figure 1a–e shows the microstructure of the five samples. In the curing procedure, the mixture of prepolymers in the light-transmitting pore would be the first region to undergo UV-initiated free radical polymerization induced by the partial effect of the photomask. The prioritized polymerization would create a concentration difference between the transmissive part and the others, causing the migration of monomers from the light mask area to the light transmission area. From Figure 1a, the polymer columns cannot obviously be observed in sample A1 containing 1 wt% C6M. Additionally, the regularity of the polymer spacer columns was optimized with the increase of C6M content. The polymer presented the grid-like microstructure in micro scale in the film-shaded area and small polymer aggregate in the light-transmissive area in sample A1 with 1 wt% content of C6M. Because the polymerization between the acrylate monomers is very fast, the radical polymerization occurred not only in the light-transmitting region, but also rapidly in the shaded region. The high viscosity due to the polymerization made it difficult for the monomer to be removed quickly into the light-transmission area. Thus, the final morphology of the polymer was detached from the shape of photomask, and the monomers spontaneously formed the phase-separated microstructure encapsulating LC droplets. As the C6M content was increased, the microstructure was gradually consistent with the photomask and the polymer space columns became more obvious. In Figure 1b, the sample A2 with 3 wt% C6M exhibits obvious polymer columns morphology, and the boundaries of polymer spacer columns were getting more pronounced as the C6M content increased, as shown in Figure 1c–e.

**Figure 2:**
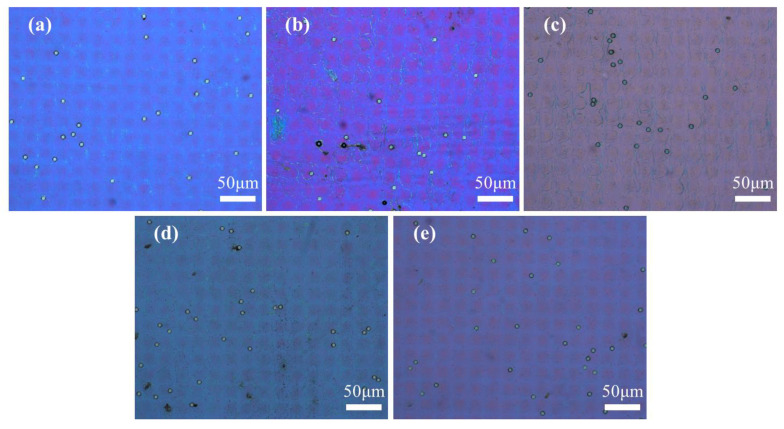
The POM image of samples A1–A5 with different contents of C6M in off state. (**a**) 1 wt%; (**b**) 2 wt%; (**c**) 3 wt%; (**d**) 4 wt%; (**e**) 5 wt%.

**Figure 3:**
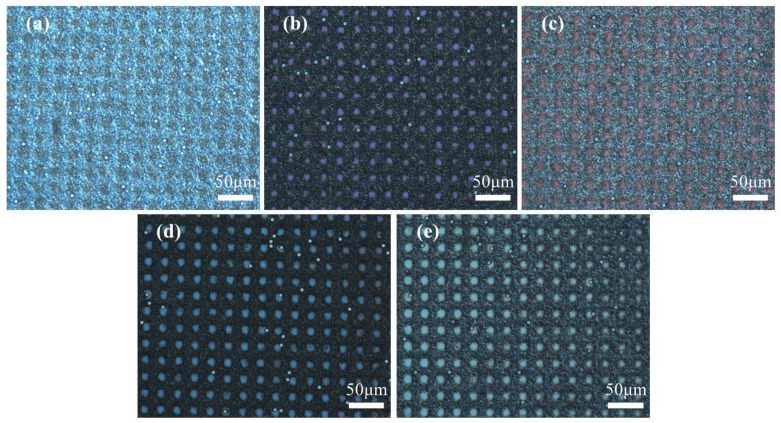
The POM image of samples A1–A5 with different contents of C6M in on state. (**a**) 1 wt%; (**b**) 2 wt%; (**c**) 3 wt%; (**d**) 4 wt%; (**e**) 5 wt%.

The POM images were taken to observe the optical texture of the samples in on and off states, and are presented in Figure 2 and Figure 3, respectively. The enlarged POM images of samples A1–A5 in on state were provided in Figure S1. The spheres in POM images are glass beads used as spacer particles to maintain the thickness of the PET substrate. The majority of CLCs in the five samples exhibited planar texture in the off state and showed the focal cone texture in the on state. From the images, the morphology of the polymer spacer columns had a great influence on the state of the LCs. When a low-frequency voltage (20 Hz) was applied across the cell, the electrohydrodynamic instability occurred and the CLCs transformed into focal cone state from initial planar state [42].

**Figure 4:**
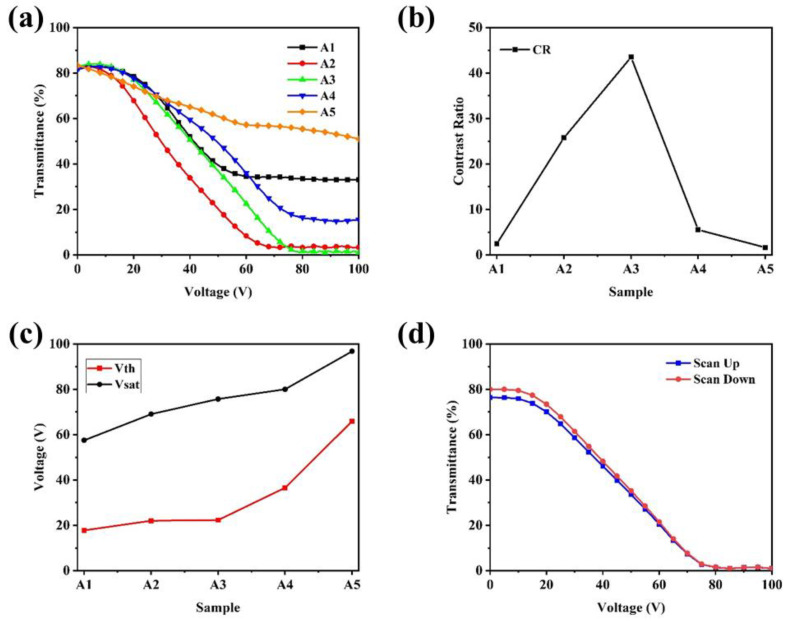
The electro-optical properties of samples A1–A5 with different contents of C6M: (**a**) voltage-transmittance curve; (**b**) CR; (**c**) *V*_th_ and *V*_sat_; (**d**) the hysteresis curves of the sample A3.

It is known that the morphology of the polymer is closely related to the behaviour of LC under electric field. Therefore, the influence of C6M content on the electro-optical properties of PSCLC samples was analysed by LCT device and illustrated in Figure 4. From the voltage-transmittance curve in Figure 4a, the samples presented obvious reverse-mode, i.e., the transmittance of the samples decreased gradually with the increase of applied voltage. The maximum transmittance (*T*_max_) occurred in the off state with no electric field, while the minimum transmittance (*T*_min_) occurred in the on state with enough electric field. As the content of C6M increased, the curve shifted to the left first and then to the right. The high transmittance of the sample A5 with excessive C6M content in the off state was contributed to the more planar texture of CLCs, consistent with the Figure 3e. From Figure 4b, the sample A3 containing 3 wt% content of C6M presented the highest contrast ratio (CR) value of 44.3, because the *T*_max_ of all the five samples were similar while the *T*_min_ was the lowest in sample A3. The CR of the samples showed a tendency of increasing first and then decreasing. In Figure 4c, the trend of *V*_th_ and *V*_sat_ increased as the C6M content increased due to the high density of polymer induced by the increasing content of C6M [43]. The hysteresis curves of the sample A3 were also analysed and plotted in Figure 4d, and the hysteresis loop was small. In conjunction with the contrast ratio and driving voltage of the five samples, the sample A3 containing 3 wt% content of C6M gave the best performance.

### 2.2. Effect of the Concentration of R811

**Figure 5:**
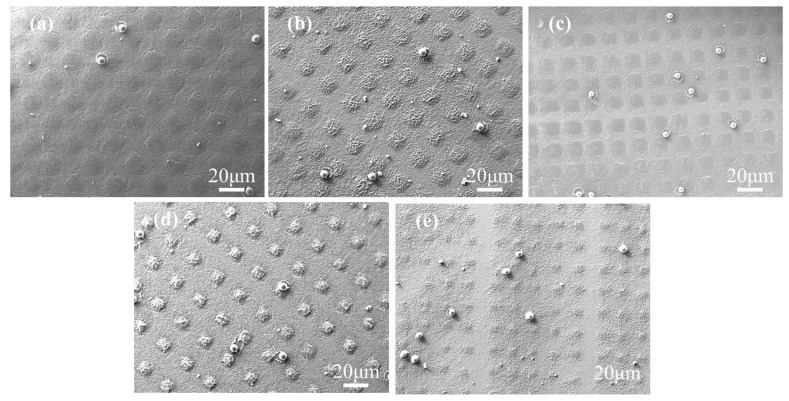
The SEM images of samples B1–B5 with different contents of R811: (**a**) 1 wt%; (**b**) 3 wt%; (**c**) 5 wt%; (**d**) 7 wt%; (**e**) 9 wt%.

In this experiment, the CLCs was composed of nematic LC and chiral dopant R811. The R811 was used to twist the nematic LC to orient with a fixed angle. The samples with 3 wt% C6M and different concentrations of R811 ranging from 1 wt% to 9 wt% were prepared, and the polymer microstructures of these samples are shown in Figure 5. The R811 is a chiral compound with a higher molecular weight, whereas LC molecule has a smaller molecular weight. Hence, the viscosity of the prepolymer mixture can be effectively increased with the increasing content of R811. The viscosity in turn affects the migration rate of monomers. In sample B1 with 1 wt% content of R811, the polymer spacer columns were clearly visible and large (Figure 5a) due to the low viscosity of the prepolymer mixture. It is noted that the higher the viscosity of the uniform polymer, the less fluid it is. Thus, the area of the polymer spacer columns decreased as the R811 content increased (Figure 5a–d). The poor molecular fluidity resulted in the difficult formation of the polymer spacer columns in sample B5 containing excessive R811 (Figure 5e).

**Figure 6:**
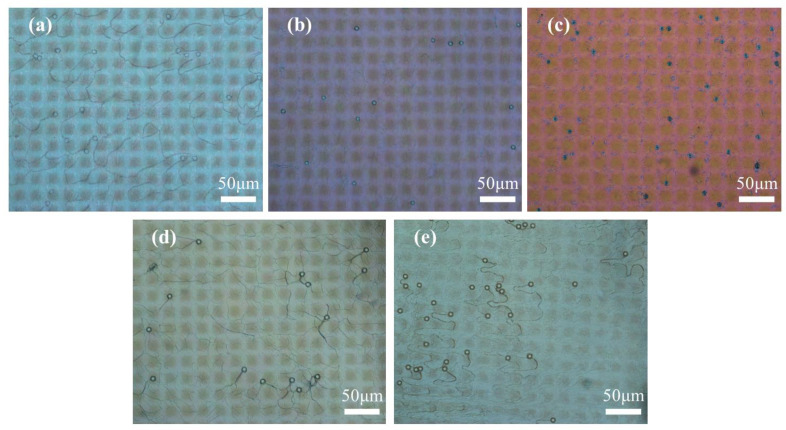
The POM image of samples B1–B5 with different contents of R811 in off state. (**a**) 1 wt%; (**b**) 3 wt%; (**c**) 5 wt%; (**d**) 7 wt%; (**e**) 9 wt%.

**Figure 7:**
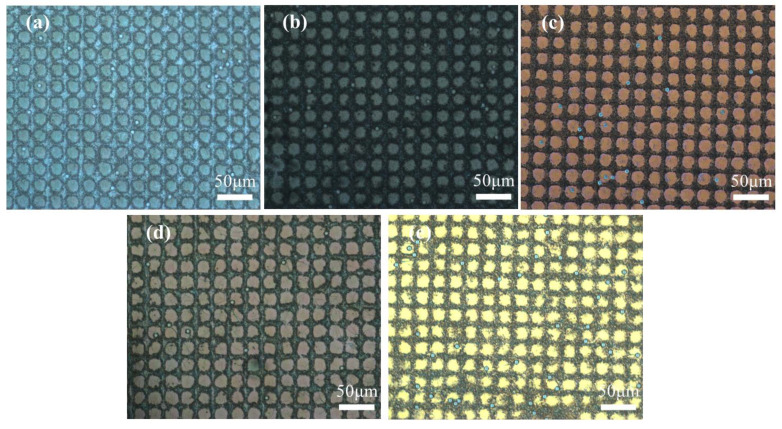
The POM image of samples B1–B5 with different contents of R811 in on state. (**a**) 1 wt%; (**b**) 3 wt%; (**c**) 5 wt%; (**d**) 7 wt%; (**e**) 9 wt%.

The optical texture of the samples containing 3 wt% C6M and different R811 concentrations in the off -state and on state are shown in Figure 6 and Figure 7, respectively. The POM picture of sample B1 with 1 wt% of R811 showed some nematic phase (Figure 6) due to the low twisting effect by low R811 concentration. With the content of R811 increased, the action of twisting was enhanced and the planar texture of the CLC gradually increased. The twisting effect became stronger with the increase of chiral dopant. Besides, the planar texture would transform to focal cone texture as the content of chiral dopant outweighed 7 wt% content of R811. When external electric field was applied to the samples, the texture of CLCs transformed into focal cone state (Figure 7 and Figure S2), and the light incident on the sample was scattered [32]. The different shape size in Figure 6 and Figure 7 may be due to the visual errors induced by light under different optical states.

**Figure 8:**
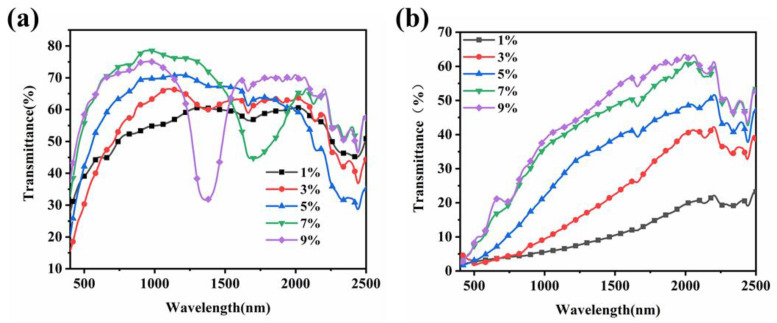
The transmittance spectra of the samples B1-B5 with different contents of R811 (**a**) without electric field and (**b**) under 60 V.

Figure 8 shows the transmittance spectra of the samples containing various contents of R811. It is noted that the CLCs could selectively reflect circularly polarized light with a wavelength equal to the pitch length. The pitch of the CLCs was defined as the periodic interlayer spacing where the liquid crystal molecule undergoes a 360° change along the helical direction and can be adjusted by varying the concentration of the chiral monomers [18]. The higher the chiral molecule content, the greater the helical twisting force provided, and the smaller the pitches of the CLCs. In the initial off state, the reflection wavelength centre blue shifted, and the wave width became narrower with increasing R811 concentration (Figure 8a), indicating that the pitch of the CLCs was shorter at higher content of R811 due to the stronger twisting forces. Additionally, the reflection wavelength centre of the samples was in the invisible near-infrared region, so the PSCLC film appeared colourless and transparent to the eye in the off state. When the samples were supplied with the electric field of 60 V (Figure 8b), the alignment of LC molecule reoriented by electric force and the transmittance of all the samples decreased. The transmittance of the samples B1–B2 containing less than 3 wt% of R811 was below 3% in the visible light. Although the transmittance of sample B3 with 5 wt% of R811 was relatively high, the average visible transmittance was still below 5%. The samples B4–B5 containing 7 wt% and 9 wt% of R811 showed an average transmittance higher than 20% in the visible region. The results indicated that the visible transmittance of the samples increased with the doping amount of the chiral molecule R811 at the same electric field.

### 2.3. Physical Picture and Bending Resistance Test of the Optimal Sample

**Figure 9:**
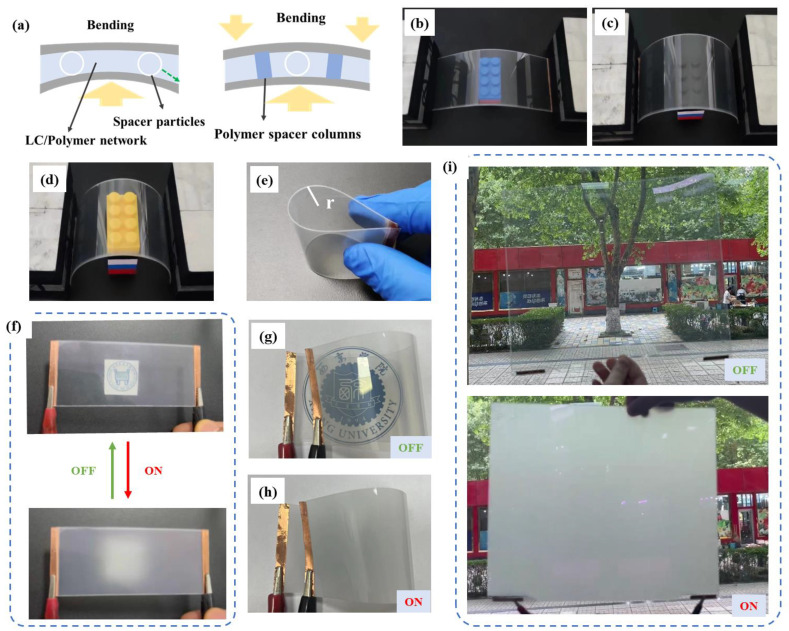
(**a**) Schematic diagram of the optimized sample with polymer columns; (**b**–**e**) photographs of film under different bending conditions; (**f**) the post-bending film photographs in off state; (**g**,**h**) the bending film photographs in off state and on state; and (**i**) the large-scale flexible film in off state and on state.

The sample B2 containing 3 wt% of C6M and 3 wt% R811 presented the best electro-optical performance and was selected for the further bending resistance test. The schematic diagram of the sample with polymer spacer columns in bending state is shown in Figure 9a. In the tiled sate, the thickness of the flexible PSCLC film was maintained by glass spacer particles. In the bending state, if there were no polymer spacer columns, the glass spacer particles would move, and the LC migrated to the thick area with the bending deformation. Thus the electro-optical properties of the flexible film were destroyed. When the polymer spacer columns were constructed, the damage caused by bending deformation would be alleviated or eliminated because of the stability of polymer spacer columns. The physical photographs of flexible film under different bending conditions are shown in Figure 9b–e. It is noted that the brightness of the film is almost the same and the coloured blocks can be clearly seen in different bending conditions. The film can be mechanically bent up to 6 mm radius curvature (Figure 9e) and kept a transparent state in that condition. Additionally, the physical pictures of the flat flexible film after bending several times in off state and on state are shown in Figure 9f. From the images, the PSCLC film exhibited high transparency in the off state, and the pattern behind the film can be clearly seen. While in the on state, the film exhibited optical scattering state, and the pattern was blocked by film when the distance between the picture and film was 1 cm. The haze of the film was 91% in the off state. The bending film photographs in off state and on state are shown in Figure 9g,h. The reversible transmittance switching by external electric field for the large-scale flexible film made by roll-to-roll process can be intuited in the Figure 9i. The electrical switch between transparent and opaque state of the PSCLC film after bending not only indicated the excellent electro-optical switching performance, but also demonstrated the outstanding bending resistance of the sample with polymer spacer columns, which makes the PSCLC film containing polymer spacer columns have a great potential to be applied in the field of flexible devices.

## 3. Experiments

### 3.1. Materials

**Figure 10:**
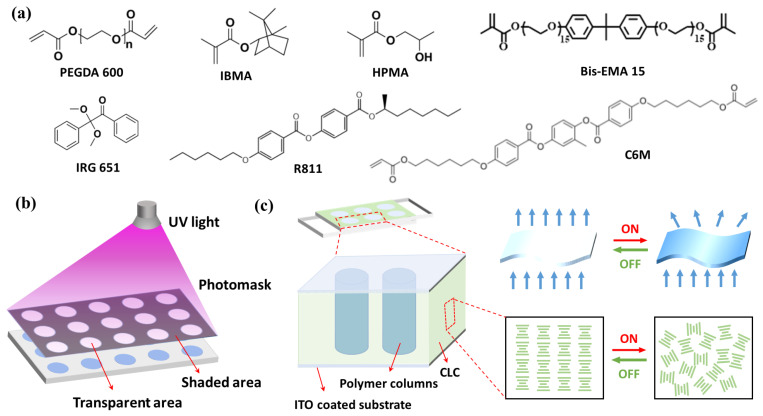
(**a**) The structures of the raw materials; (**b**) Schematic illustration for the fabrication process; and (**c**) Schematic diagram of the film operation.

In this experiment, the SLC 3MV-8173 (*T*_NI_ = 98.2 °C, Δ*n* = 0.18, Δ*ε* = −7.5, Jiangsu Synthesis Display Technology Co., Ltd., Nanjing, China) was used as negative anisotropic LC, and the R811 (HTP ≈ 11.3 μm^−1^, Shijiazhuang Yesheng Chemical Technology Co., Ltd., Shijiazhuang, China) was used as chiral additive. The non-liquid crystal monomers (NLCM), composed of polyethylene glycol diacrylate (PEGDA 600, 98%), hydroxypropyl methacrylate (HPMA, 97%), isobornyl methacrylate (IBMA, 98%), and Bis-EMA 15 (98%), and the liquid crystal monomer (LCM), 2-methyl-1,4-phenyl-bis[4-(6-arcyloyoxyhexyloxy) benzoate] (C6M, 98%) were purchased from Shanghai Macklin Biochemical Technology Co., Ltd. (Shanghai, China). The IRG651 was used as free radical photo-initiator. The chemical structures of raw materials are displayed in Figure 10a. The light-transmitting holes in the photomask (Anhui Huateng Optoelectronics Technology Co., Ltd., Hefei, China) were 8 μm in diameter.

### 3.2. Sample Preparation

Prior to the experiment, two polyethylene terephthalate (PET) substrates coated with ITO were glued together to prepare an LC cell with a thickness of 20 μm by glasses beads. The cholesteric LCs phase was prepared first by mixing nematic SLC 3MV-8173 and chiral centres R811 in a certain ratio. Then, the above CLCs, acrylate polymerizable monomers, 20 μm glass beads, and IRG 651 were vigorously stirred into a homogeneous solution. Subsequently, the blend was dripped into an LC cell by roll-to-roll process. The components of all the samples in this experiment are summarized in Table 1. Finally, the photomask was placed on the top of the LC cell (Figure 10b), and the polymerization of acrylate monomers was triggered with ultraviolet light (365 nm, 3.0 mW/cm^2^) for 10 min at room temperature. After removing the photomask, the LC cell was directly exposed to UV light for 5 min to ensure complete polymerization. During the polymerization process, a high-frequency electric field of 100 V at 1 kHz was applied to the film to ensure the planar state of the CLCs. The schematic diagram of the film operation is shown in Figure 10c.

### 3.3. Characterization

The optical textures of the samples were characterized by polarizing optical microscope (POM, Carl Zeiss, AxioVision SE64, Jena, Germany). The microstructures of the polymer matrix were observed under scanning electron microscopy (SEM, HITACHI S-4800, Tokyo, Japan). Before the observation, all the samples were immersed in cyclohexane for 2 weeks to completely remove LC molecules. After they were dried in a vacuum oven at 60 °C for 2 h, the samples were treated with gold sputtering. The optical modulation performance of the samples in visible wavelength was tested by UV/Vis/NIR spectrometer (PerkinElmer Lambda 950, Waltham, MA, USA). The transmittance of an empty LC cell was normalized as 100%. The electro-optical performances of the samples were analysed by a comprehensive LC parameter tester (LCT-5016C, Beijing Liquid Crystal Engineering Research and Development Center, Beijing, China). The incident light emitter wavelength of the tungsten halogen lamp was 560 nm. The distance between the sample and the photodiode was controlled at 30.0 cm. The wavelength of the driving electric field is a sinusoidal wave. The threshold voltage (*V*_th_) and saturation voltage (*V*_sat_) are denoted as the voltages that enable the transmittance of the sample to achieve 10% and 90% of the saturation level, respectively. The maximum and minimum transmittance of the sample are denoted as *T*_max_ and *T*_min_, respectively. The contrast ratio (CR) was calculated by *T*_max_/*T*_min_.

## 4. Conclusions

In this work, the reverse-mode PSCLC flexible film with polymer spacer columns was prepared by the employment of photomask. The electro-optical properties and the microstructure of the polymer can be regulated by the concentration of C6M and R811. The experimental results indicated that the microstructure was gradually consistence with the photomask and the boundaries of polymer spacer columns were getting more pronounced as the C6M content increased. By applying a low-frequency electric field, the initial planar state of the sample transformed into focal cone state. The CR of the samples containing different content of C6M showed a tendency of increasing first and then decreasing. The area of polymer spacer columns decreased when the R811 content increased. The sample B2 containing 3 wt% of C6M and 3 wt% R811 presented the best electro-optical performance. The flexible PSCLC film with polymer spacer columns presented outstanding bending resistance, which makes it have a great potential to be applied in the field of flexible devices.

## Figures and Tables

**Table 1:** The composition of the samples.

Samples ^a^	CLC		Pitch (μm)	NLCM (wt%) ^b^	C6M (wt%)	IRG 651
	SLC 3MV-8173	R811				
Group Ⅰ						
A1	91	5	1.44	3	1	0.5
A2	90	5	1.43	3	2	0.5
A3	89	5	1.41	3	3	0.5
A4	88	5	1.40	3	4	0.5
A5	87	5	1.38	3	5	0.5
Group Ⅱ						
B1	93	1	7.07	3	3	0.5
B2	91	3	2.36	3	3	0.5
B3	89	5	1.41	3	3	0.5
B4	87	7	1.01	3	3	0.5
B5	85	9	0.79	3	3	0.5

^a^ The acylate mixture is composed of PEGDA 600 (10 wt%), HPMA (10 wt%), Bis-EMA 15 (10 wt%), and IBMA (70 wt%). ^b^ The content of IRG 651 is relative to the total weight.

## Data Availability

The data that support the findings of this study are available from the corresponding author upon reasonable request.

## Supplementary Materials

The following supporting information can be downloaded at: https://www.mdpi.com/article/10.3390/molecules29174276/s1, Figure S1: The enlarged POM image of samples A1–A5 with different content of C6M in on state. Figure S2: The enlarged POM image of samples B1–B5 with different content of R811 in on state.
