# Supplementary material for: Reverse Mode Polymer Stabilized Cholesteric Liquid Crystal Flexible Films with Excellent Bending Resistance
Source: Molecules. 2024 Sep 9;29(17):4276. doi: 10.3390/molecules29174276 (PMC11397460; doi:10.3390/molecules29174276)
Supplement: Supplementary file 1 [file molecules-29-04276-s001.zip › molecules-3139401-supplementary.pdf]

Supplementary Information (SI)

Reverse Mode Polymer Stabilized Cholesteric Liquid Crystal Flexible Films with Excellent Bending Resistance

Ping Yu [1], Zemin He [1,*], Yuzhen Zhao [1], Wenqi Song [1] and Zongcheng Miao [2,*]

1 Technological Institute of Materials & Energy Science (TIMES), Xi'an Key Laboratory of Advanced Photo-Electronics Materials and Energy Conversion Device, School of Electronic Information, Xijing University, Xi'an 710123, China.

2 School of Artificial Intelligence, Optics and Electronics (IOPEN), Northwestern Polytechnical University, Xi'an 710072, China.

* Correspondence: zeminhe315@126.com (Z.H.); miaozongcheng@nwpu.edu.cn (Z.M.)

Content:

Figure S1. The enlarged POM image of samples A1-A5 with different content of C6M in on state.
Figure S2. The enlarged POM image of samples B1-B5 with different content of R811 in on state.

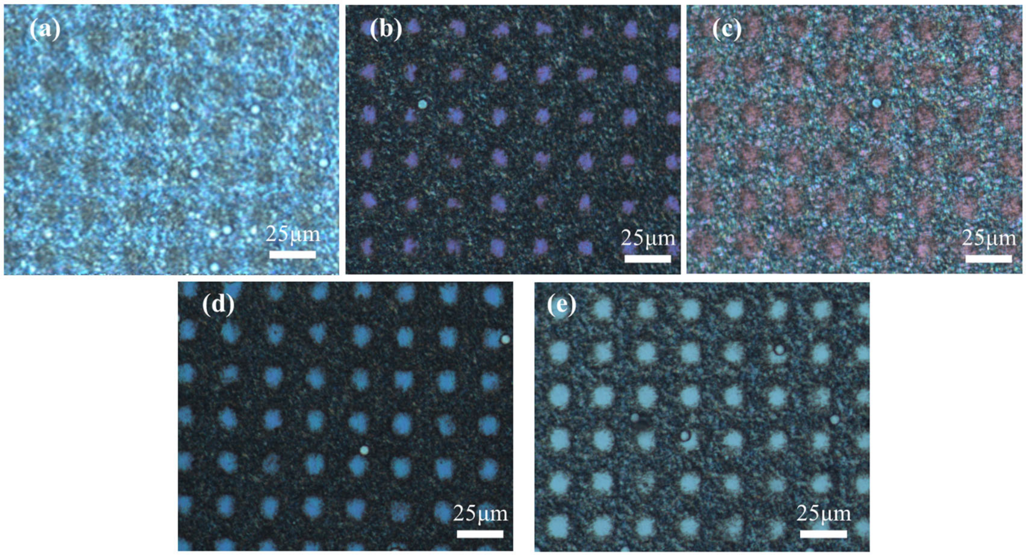

Figure S1. The enlarged POM image of samples A1-A5 with different content of C6M in on state.

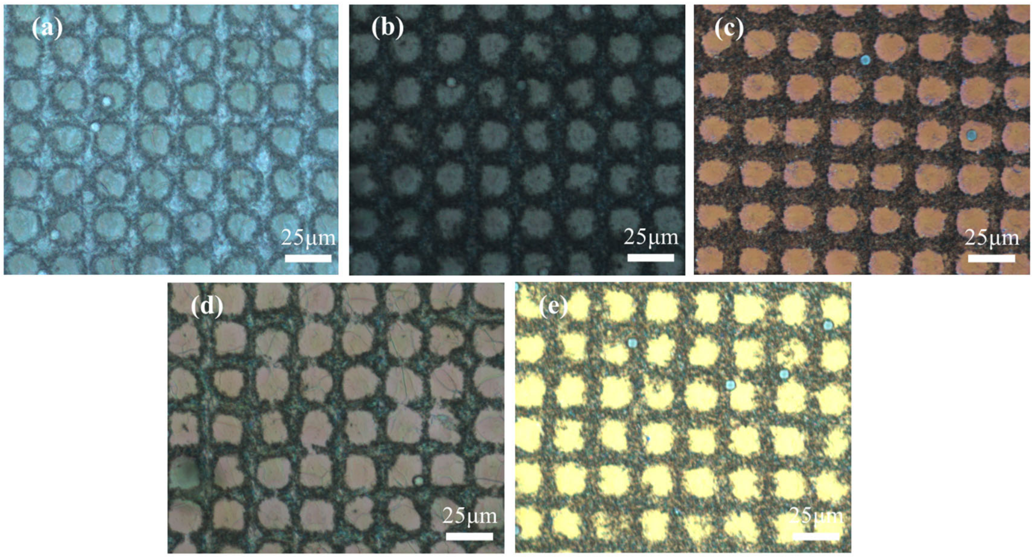

Figure S2. The enlarged POM image of samples B1-B5 with different content of R811 in on state.
